# Rapamycin Upregulates Connective Tissue Growth Factor Expression in Hepatic Progenitor Cells Through TGF-β-Smad2 Dependent Signaling

**DOI:** 10.3389/fphar.2018.00877

**Published:** 2018-08-08

**Authors:** Yu Wu, Wei Wang, Xiang-mei Peng, Yi He, Yi-xiao Xiong, Hui-fang Liang, Liang Chu, Bi-xiang Zhang, Ze-yang Ding, Xiao-ping Chen

**Affiliations:** ^1^Hepatic Surgery Center, Tongji Hospital, Tongji Medical College, Huazhong University of Science and Technology, Wuhan, China; ^2^Department of Nephrology, Liyuan Hospital, Tongji Medical College, Huazhong University of Science and Technology, Wuhan, China

**Keywords:** rapamycin, liver fibrosis, hepatic progenitor cells, transforming growth factor-β, connective tissue growth factor

## Abstract

Rapamycin (sirolimus) is a mTOR kinase inhibitor and is widely used as an immunosuppressive drug to prevent graft rejection in organ transplantation currently. However, some recent investigations have reported that it had profibrotic effect in the progression of organ fibrosis, and its precise role in the liver fibrosis is still poorly understood. Here we showed that rapamycin upregulated connective tissue growth factor (CTGF) expression at the transcriptional level in hepatic progenitor cells (HPCs). Using lentivirus-mediated small hairpin RNA (shRNA) we demonstrated that knockdown of mTOR, Raptor, or Rictor mimicked the effect of rapamycin treatment. Mechanistically, inhibition of mTOR activity with rapamycin resulted in a hyperactive PI3K-Akt pathway, whereas this activation inhibited the expression of CTGF in HPCs. Besides, rapamycin activated the TGF-β-Smad signaling, and TGF-β receptor type I (TGFβRI) serine/threonine kinase inhibitors completely blocked the effects of rapamycin on HPCs. Moreover, Smad2 was involved in the induction of CTGF through rapamycin-activated TGF-β-Smad signaling as knockdown completely blocked CTGF induction, while knockdown of Smad4 expression partially inhibited induction, whereas Smad3 knockdown had no effect. Rapamycin also induced ROS generation and latent TGF-β activation which contributed to TGF-β-Smad signaling. In conclusion, this study demonstrates that rapamycin upregulates CTGF in HPCs and suggests that rapamycin has potential fibrotic effect in liver.

## Introduction

Liver fibrosis is regarded as an imbalanced tissue repair response with excessive accumulation of extracellular matrix proteins in response to chronic liver injury (Bataller and Brenner, 2005; Pellicoro et al., 2014; Weiskirchen et al., 2018). Persistent liver fibrosis results in cirrhosis which may lead to portal hypertension, end-stage liver disease, or the initiation of HCC (Zhang and Friedman, 2012). Indeed, almost 80-90% of HCC cases arises in cirrhotic liver (Fattovich et al., 2004). In fibrotic liver, hepatocyte-mediated regeneration is usually impaired and subsequently triggers activation of the progenitor (oval) cell compartment, which in turn provokes a severe fibrogenic response (Tirnitz-Parker et al., 2014). Activation of HPCs serves as an alternative regeneration pathway when the replicative capacity of hepatocytes is impaired. Previous studies have shown that activated HPCs participate in the progression of liver fibrosis, and that the degree of progenitor cell activation is directly proportional to the severity of fibrosis (Lowes et al., 1999; Clouston et al., 2005). CTGF is a matricellular protein strongly upregulated in fibrotic liver tissue, and it plays a pivotal role in fibrogenesis of liver (Gressner and Gressner, 2008; Weiskirchen, 2016). Previous investigations have demonstrated that hepatocytes, cholangiocytes, and HSCs, as well as HPCs express and secrete CTGF in the fibrotic liver (Gressner et al., 2007; Ding et al., 2013, 2016; Williams et al., 2014).

For patients with end-stage liver disease or HCC, liver transplantation is regarded as the definitive therapy and rapamycin (sirolimus) is widely used in the antirejection treatment after transplantation (Asrani et al., 2010; Kawahara et al., 2011). Rapamycin was first approved for post kidney transplantation therapy by the US FDA in 1999 (Miller, 1999). Owing to its potent immunosuppressive activity and reduced kidney toxicity compared to CNIs, rapamycin was soon approved as an immunosuppressive alternative which is increasingly used to eliminate or at least lower CNIs-induced nephrotoxicity (Kawahara et al., 2011). Rapamycin exerts its function by forming a complex with its cellular receptor FKBP12 (FK506-binding protein of 12 kDa), which blocks interactions between mTOR, an evolutionarily conserved serine/threonine kinase, and regulatory proteins (Yang et al., 2013). In addition to immune modulation, mTOR regulates both cell growth and metabolism by acting as an integrator of nutrients (amino acids and energy) and growth factors (Shamji et al., 2003).

Independent of its immunosuppressive action, rapamycin has shown controversial roles in organ fibrosis. Rapamycin was reported to reduce renal interstitial fibrosis by diminishing the number of interstitial fibroblasts and myofibroblasts in a rodent model of renal fibrosis (Wang et al., 2010). Similarly, Wang et al. (2015) found that rapamycin attenuated aldosterone-induced tubulointerstitial inflammation and fibrosis by blocking mTOR signaling. Conversely, Osman et al. (2009) reported that rapamycin induced rapid activation of the fibrogenic Smad signaling cascade and upregulated CTGF and plasminogen activator inhibitor 1 (PAI-1) expression in rat mesangial cells. Further, rapamycin augmented CTGF expression in kidney tissue and promoted kidney fibrosis in a rat model of chronic nephrotoxicity (Shihab et al., 2006). High doses of everolimus may also induce renal fibrosis by activating epithelial to mesenchymal transition (EMT) of renal tubular cells (Masola et al., 2013). Clinical results in renal transplantation are also inconsistent. Pontrelli et al. (2008) reported that rapamycin could reduce interstitial fibrosis in chronic allograft nephropathy after renal transplantation, whereas Servais et al. (2009) did not find a significant reduction in interstitial fibrosis at 1 year after renal transplantation in patients converted from cyclosporine (CsA)-based to rapamycin-based therapy. Further, these discordant effects have been observed in pulmonary fibrosis. Rapamycin showed anti-fibrotic effects against transforming growth factor α (TGF-α) and TGF-β-induced pulmonary fibrosis in rats (Korfhagen et al., 2009; Gao et al., 2013), and both rapamycin and rapamycin analog SDZ RAD attenuated bleomycin-induced pulmonary fibrosis in rats (Simler et al., 2002; Tulek et al., 2011), but Xu et al. (2013, 2015) found that rapamycin increased CTGF expression of lung fibroblasts and epithelial cells via PI3K activation. Tomei et al. (2016) also revealed the profibrotic effect of everolimus by inducing EMT in bronchial/pulmonary cells. These discrepancies may reflect differential effects of rapamycin among fibrogenic cells and factors involved in different fibrosis models. In other words, roles of rapamycin in fibrosis may be cell context dependent.

Currently, studies on the relationship between rapamycin treatment and liver fibrosis are limited and as in other systems, results are controversial. Previous studies reported that rapamycin attenuated hepatic fibrosis in carbon tetrachloride and BDL models (Zhu et al., 1999; Biecker et al., 2005; Neef et al., 2006). The rapalogs everolimus also showed anti-fibrotic activity in BDL model (Patsenker et al., 2011). However, another investigation showed that rapamycin did not attenuate the progression of liver fibrosis (Renken et al., 2011). High doses of everolimus seemed to have profibrotic activity by inducing EMT in HSC and HepG2 cells (Masola et al., 2015). These results suggest that the precise role and the underlying molecular mechanism of rapamycin in liver fibrosis remain poorly understood.

In this study, we showed that rapamycin upregulated CTGF expression in HPCs. Mechanistically, inhibition of mTOR activity with rapamycin resulted in a hyperactive PI3K-Akt pathway, whereas this activation inhibited the expression of CTGF in HPCs. Besides, rapamycin increased ROS generation and subsequently activated TGF-β-Smad2 signaling to promote CTGF expression.

## Materials and Methods

### Reagents and Antibodies

Reagents were obtained from the following sources: rapamycin was from Selleckchem (Houston, TX, United States). SB431542, LY364947, LY294002, U0126, SP600125, and SB203580 were from Cayman (Ann Arbor, MI, United States). NAC was from TCI (Tokyo, Japan). DCFH-DA was from Sigma-Aldrich (St. Louis, MO, United States). Cycloheximide was from Beyotime Institute of Biotechnology (Haimen, Jiangsu, China).

Primary antibodies: CTGF (L-20) antibody (sc-14939), Smad2/3 (C-8) antibody (sc-133098) were obtained from Santa Cruz Biotechnology (Santa Cruz, CA, United States). Phospho-Smad2 (Ser465/467) antibody (#3108), Smad2 antibody (#5339), Smad3 antibody (#9523), β-Actin antibody (#4967), Phospho-mTOR (Ser2448) antibody (#5536), 4E-BP1 antibody (#9644), Phospho-4E-BP1 (Thr37/46) antibody (#2855), Phospho-p70 S6 Kinase (Thr389) antibody (#9206), p70 S6 Kinase antibody (#2708), Phospho-Akt (Ser473) antibody (#4060), Akt (pan) antibody (#4691), Raptor antibody (#2280), Rictor antibody (#2114), TGF-β antibody (#3709) were obtained from Cell Signaling Technology (Beverly, MA, United States). Phospho-Smad3 (Ser423/425) antibody (1880-1), Smad4 antibody (1676-1) were obtained from Epitomics (Burlingame, CA, United States). mTOR antibody (ab134903) were obtained from Abcam (Cambridge, United Kingdom).

Secondary antibodies: HRP conjugated anti-rabbit IgG, HRP conjugated anti-mouse IgG, HRP conjugated anti-goat IgG, Cy3-conjugated goat anti-mouse IgG were purchase from Jackson ImmunoResearch Laboratories (West Grove, PA, United States).

### Cell Culture

Hepatic progenitor cell line LE6 was a generous gift from Dr Nelson Fausto. WB-F344 cells were purchased from Shanghai Cell Bank and Department of Pathology of Second Military Medical University (Shanghai). Both of them have proven to be useful *in vitro* models for studying functions of HPCs (Nguyen et al., 2007; Duncan et al., 2009; Ding et al., 2013, 2016). The lentivirus packaging cell line 293T, HCC cell lines HepG2, Hep3B, and SMMC-7721 were purchased from China Center for Type Culture Collection (CCTCC, Wuhan, China). HCCLM3 were provided by Liver Cancer Institute, Zhongshan Hospital, Fudan University (Shanghai, China). The cell line from human noncancerous liver tissue QSG-7701 and rat liver cell line BRL were obtained from cell bank of Chinese Academy of Sciences (Shanghai, China). LE6 cell was cultured in Dulbecco’s modified Eagle’s medium : Ham’s F-10 (1:1) (Gibco Laboratories) supplemented with 10% fetal bovine serum (FBS, Gibco Laboratories), 1 μg/ml insulin, 0.5 μg/ml hydrocortisone (Braun et al., 1987). Cells were incubated at least 12 h in serum free media for serum starvation before their use in experiments. The 293T, HepG2, Hep3B SMMC-7721, HCCLM3, QSG-7701, and BRL cells were maintained in DMEM medium supplied with 10% FBS.

### RNA Purification and Quantitative RT-PCR

Total RNA was isolated from LE/6 cell using the TRIzol reagent (Invitrogen, Carlsbad, CA, United States). Two milligram of RNA from each sample was reverse-transcribed with the FastQuant RT Kit (With gDNase) (KR106) (Tiangen, Beijing, China). Real time polymerase chain reaction (PCR) was performed with an ABI ViiA 7 Dx instrument (Applied Biosystems, Foster City, CA, United States) using SuperReal PreMix Plus (SYBR Green) (FP205) PCR reagents (Tiangen, Beijing, China). The fold changes of the target genes were calculated using the 2^-ΔΔCT^ method. The CTGF primers used for PCR reactions were: forward sequence, 5′- TAGCTGCCTACCGACTGGAA -3′; reverse sequence, 5′- CTTAGAACAGGCGCTCCACT -3′. The GAPDH primers used were: forward sequence, 5′- AGACAGCCGCATCTTCTTGT -3′; reverse sequence, 5′- CTTGCCGTGGGTAGAGTCAT -3′.

### Immunoblotting

Cells were harvested and lysed with radioimmunoprecipitation assay (RIPA) (Pierce, Rockford, IL, United States) buffer supplied with cOmplete^TM^, Mini, EDTA-free Protease Inhibitor Cocktail and PhosSTOP^TM^ phosphatase inhibitor tablets (Roche, Basel, Switzerland). After centrifugation at 12,000 rpm, 4°C for 15 min to pellet cell debris, protein concentrations were determined using the Bicinchoninic acid assay kit (Pierce, Rockford, IL, United States). Equal amount of protein samples (40 μg) were resolved in 8–10% sodium dodecyl sulfate-polyacrylamide gel electrophoresis (SDS-PAGE) and transferred onto 0.45 μm polyvinylidene difluoride (PVDF) membranes (Millipore, Billerica, MA, United States). Membranes were subsequently blocked with 5% nonfat dry milk or bovine serum albumin (BSA) in 1X TBST (20 mM Tris-HCl, pH7.6, 150 mM Sodium Chloride, 0.1%Tween-20) for 1 h at room temperature. Primary antibody was incubated overnight at 4°C with gentle shaking. After washing in TBST three cycles for 5 min each, membranes were incubated with horseradish peroxidase-conjugated secondary antibody for 1 h at room temperature. Immunodetection was performed using Clarity^TM^ Western ECL Substrate (Bio-Rad, Hercules, CA, United States) with ChemiDoc^TM^ XRS+ Imaging System (Bio-Rad, Hercules, CA, United States).

### Immunofluorescence

Cells were seeded and cultured in a 24-well plate. After treatments cells were fixed with 4% paraformaldehyde for 15 min at room temperature and permeabilized in 0.2% TritonX-100 solution (dissolved in PBS) for 15 min. Block specimen in normal serum from the same species as the secondary antibody for 30 min at room temperature. Then, cells were incubated overnight at 4°C in the diluted Smad2/3 antibody in 1% BSA in PBST in a humidified chamber. Cy3-conjugated anti-mouse IgG was used to incubate cells for 1 h at room temperature in the dark. Nucleus were stained by DAPI (Wuhan Promoter Biotechnology Co., Ltd., Wuhan, China). Images were taken by EVOS^TM^ FL Imaging System (Thermo Fisher Scientific, Waltham, MA, United States).

### Plasmids

pLKO.1-TRC cloning vector was a gift from David Root (Addgene plasmid # 10878) (Moffat et al., 2006). pRSV-Rev was a gift from Didier Trono (Addgene plasmid # 12253)(Dull et al., 1998). pMDLg/pRRE was a gift from Didier Trono (Addgene plasmid # 12251) (Dull et al., 1998). pMD2.G was a gift from Didier Trono (Addgene plasmid # 12259). SBE4-luc (Addgene plasmid 16495) was a gift from Bert Vogelstein (Johns Hopkins Kimmel Cancer Center, Baltimore, MD, United States) (Zawel et al., 1998). pRL-TK was purchased from Promega (Madison, WI, United States). CTGF-luc reporter plasmid was constructed as described previously (Ding et al., 2013).

To create pLKO.1-shRNA plasmids, double-stranded oligonucleotides shRNA fragments were chemically synthesized by Tsingke biological technology (Beijing, China), and were cloned into AgeI/EcoRI site of the pLKO.1-TRC cloning vector. shRNA sequences for each individual shRNA are as follows: shmTOR#1: ATGCTGTCCCTGGTCCTTATG; shmTOR#2: CAAGGCTTCTTCCGTTCTATC; shRaptor#1: GCTGCAATTAACCCAAACCAT; shRaptor#2: CC TCATCGTCAAGTCCTTCAA; shRictor#1: GCCATCTGAATA ACTTCACAA; shRictor#2: AAGACGAGCCACTATCTGACA; shSmad2: GCCAGTTACTTATTCAGAACCTGCA; shSmad3: CTGTCCAATGTTAACCGGAAT; shSmad4: CAGCTACTTACCACCATAACA; shScramble: CCTAAGGTTAAGTCGCCCTCG.

### Lentivirus Production

Lentivirals were produced by co-transfection of 293T cells with pLKO.1-shRNA, pRSV-Rev, pMDLg/pRRE, and pMD2.G using Lipofectamine 3000 (Thermo Fisher Scientific, Waltham, MA, United States) according to the manufacturer’s instructions. Cells were transfected for 12–15 h, and then changed media to DMEM/20% FBS. Lentiviral supernatants were collected and filtered through a 0.45 μm filter (Millipore, Billerica, MA, United States) after incubate for additional 48 h. Cells were infected with the lentivirus and selected with puromycin (5 μg/mL).

### Adenovirus Construction and Infection

Rat Smad3 overexpression adenovirus were obtained from the Hanbio Co., Ltd. (Shanghai, China). Cells were seed in 6-well plate at 30∼40% confluence. The next day, cells were changed with 1 mL fresh media and added equivalent Ad-GFP and Ad-Smad3 adenovirus. After 1 h incubation, cells were supplied with 1 mL media with 5 μg/mL polybrene. Change fresh media 24 h later and allow for growing another 72 h. Infection efficiency were visualized by GFP expression. Then cells were used for the experiments.

### Dual-Luciferase Reporter Assay

Cells were seeded in 24-well plate at a density of 5 × 10^4^ cells per well. The next day, cells were co-transfected with 0.48 μg promoter reporter plasmids and 0.02 μg pRL-TK plasmids. Transfections were performed using Lipofectamine 3000 (Thermo Fisher Scientific, Waltham, MA, United States) according to the manufacturer’s instructions. At 6 h after transfection, cells were replaced with fresh medium and allowed to growth for 24 h. Serum-starved cells were used for the assay. Luciferase activities were detected with the Dual-Luciferase Reporter Assay System (Promega, Madison, WI, United States) using a GloMax 20/20 Luminometer (Promega, Madison, WI, United States) according to the manufacturer’s instructions. Firefly luciferase activity was normalized to Renilla activity.

### ROS Detection Assay

Detection of the generation of ROS was done by using a cell-permeable fluorimetric probe named 2′,7′-DCFH-DA. DCFH-DA crosses the cell membrane and is de-esterified into 2′,7′-DCFH intracellularly. The resulting DCFH reacts with intracellular ROS to give the highly fluorescent 2′,7′-DCF. Serum-starved cells were treated with rapamycin (10 nM) in the absence or presence of NAC (5 mM) for 1 h. Then, cells were washed once with PBS and incubated in the dark at 37°C for 30 min in 10 μM DCFH-DA. After washed with FBS free media three times, ROS generation was visualized by fluorescent DCF formation using EVOS^TM^ FL Imaging System (Thermo Fisher Scientific, Waltham, MA, United States). Images were quantified by image pro plus (IPP) software.

### Statistical Analysis

For statistical analysis, SPSS 22.0 (SPSS, Chicago, IL, United States) was used. Statistical analyses were carried out by two-tailed unpaired Student’s *t*-test and one-way analysis of variance (ANOVA) as appropriate. Values of *p* < 0.05 were considered statistically significance.

## Results

### Rapamycin Induces *de novo* Synthesis of CTGF in HPCs

**FIGURE 1:**
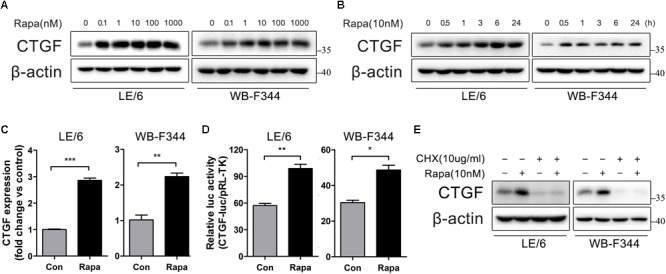
Rapamycin induces *de novo* synthesis of CTGF in HPCs. **(A)** LE/6 and WB-F344 cells was treated with Rapamycin at indicated concentrations for 6 h. Lysates were subjected to Western blot analysis with antibodies against CTGF. β-actin was used as a loading control; **(B)** LE/6 and WB-F344 cells was stimulated with rapamycin (10 nM) for the indicated times before cells were harvested for immunoblotting analysis against CTGF. β-actin was used as a loading control; **(C)** LE/6 and WB-F344 cells was treated with Rapamycin (10 nM) for 6 h, and the relative CTGF expression was analyzed by qRT-PCR. Result was means ± SD of triplicate measurements. Experiment was repeated three times; ^∗∗^*p* < 0.01, ^∗∗∗^*p* < 0.001; **(D)** LE/6 and WB-F344 cells was co-transfected with pRL-TK and CTGF-luc plasmids and then treated with Rapamycin for 16 h. Luciferase activity was normalized to renilla luciferase activity. Results showed as means ± SD of triplicate measurements. ^∗^*p* < 0.05, ^∗∗^*p* < 0.01 compared with the control; **(E)** LE/6 and WB-F344 cells was treated with Rapamycin (10 nM) and cycloheximide (10 μg/mL) as indicated for 6 h. Lysates were subjected to Western blot analysis with antibodies against indicated proteins. β-actin was used as a loading control.

To investigate the effects of rapamycin on fibrogenesis, we first treated cultured liver cell lines (BRL and QSG-7701) with rapamycin and measured the expression of CTGF, which is regarded as “the master switch” in liver fibrosis (Gressner and Gressner, 2008). However, western blotting showed no effect of rapamycin on the expression of CTGF in either BRL or QSG-7701 cells (**Supplementary Figures 1A,B**). Considering that diverse types of cells participate in the progression of liver fibrosis, and HPCs may play a particularly important role, we then investigated whether rapamycin modulates CTGF expression in two HPCs LE/6 and WB-F344 cells which were widely used as *in vitro* models for studying functions of HPCs (Nguyen et al., 2007; Ding et al., 2013, 2016; Wu et al., 2018). Western blotting revealed that rapamycin upregulated CTGF protein expression in both LE/6 and WB-F344 cells in a dose and time dependent manner (**Figures 1A,B**). Real-time PCR analyses further demonstrated that 10 nM rapamycin significantly elevated CTGF mRNA level in LE/6 and WB-F344 cell cultures by 2.86 ± 0.15 and 2.24 ± 0.17 fold, respectively (*p* < 0.05, **Figure 1C**). Luciferase reporter assays further demonstrated that rapamycin upregulated CTGF at the transcriptional level (57.12 ± 4.09 vs 98.89 ± 8.03 in LE/6, *p* < 0.01; 30.42 ± 1.79 vs 48.67 ± 3.76 in WB-F344, *p* < 0.05) (**Figure 1D**). Treatment with cycloheximide, a eukaryote protein synthesis inhibitor, also blocked rapamycin-induced CTGF production in HPCs (**Figure 1E**). We also tested the effect of rapamycin on hepatoma-derived cell lines HepG2, Hep3B, SMMC-7721, and LM3 cells but found no effect on CTGF expression (**Supplementary Figures 1C–F**). These results suggest that rapamycin induces *de novo* synthesis of CTGF in HPCs.

### Rapamycin Attenuates the Activity of mTOR Signaling, Which Contributes to CTGF Induction in HPCs

**FIGURE 2:**
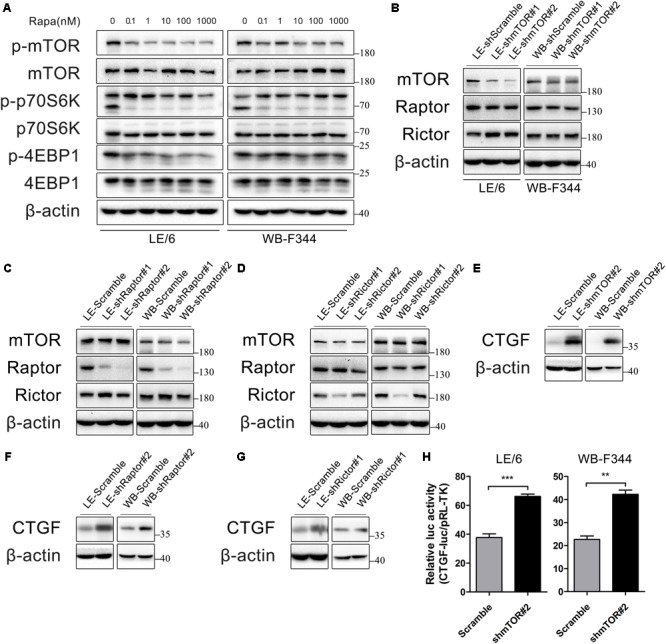
Rapamycin attenuates the activity of mTOR signaling, which contributes to CTGF induction in HPCs. **(A)** LE/6 and WB-F344 cells were treated with rapamycin at indicated concentrations for 6 h. Lysates were subjected to Western blot analysis with antibodies against indicated proteins. β-actin was used as a loading control; **(B–G)** LE/6 and WB-F344 cells were transfected with lentivirus carrying shRNA against mTOR, Raptor, Rictor, or scramble shRNA and Western blot analysis showed the expression of these proteins. β-actin was used as a loading control; **(H)** LE-shmTOR#2, WB-shmTOR#2 and their scramble control (shScramble) were co-transfected with pRL-TK and CTGF-luc plasmids for 24 h. Luciferase activity was normalized to renilla luciferase activity. Results showed as means ± SD of triplicate measurements. ^∗∗^*p* < 0.01, ^∗∗∗^*p* < 0.001 compared with the control.

Rapamycin exerts its function through inhibition of mTOR signaling. Therefore, we confirmed the contribution of mTOR signaling to rapamycin-induced CTGF upregulation in HPCs. The primary downstream targets of mTOR are p70S6 kinase 1 (p70S6K1) and eukaryotic initiation factor 4E binding protein 1 (4E-BP1) (Wullschleger et al., 2006). We initially examined whether mTOR-p70S6K/4EBP1 signaling responded to rapamycin in HPCs. As shown in **Figure 2A**, rapamycin (0.1–1000 nM) significantly decreased phosphorylation of mTOR, p70S6K, and 4EBP1 even at 0.1 nM.

mTOR forms two distinct protein complexes, mTOR Complex 1 (mTORC1) and 2 (mTORC2). Proteins mLST8 (mammalian lethal with Sec13 protein 8, also known as GßL) and DEPTOR (DEP domain containing mTOR interacting protein) exist in both mTORC1 and mTORC2. mTORC1 is characterized by Raptor (regulatory protein associated with mTOR) and PRAS40 (proline-rich Akt substrate of 40 kDa) while Rictor (rapamycin insensitive companion of mTOR), mSin1, and Protor1/2 are specific to mTORC2 (Saxton and Sabatini, 2017). Knockdown of mTOR impairs both mTORC1 and mTORC2 activity, while knockdown of Raptor and Rictor ablate mTORC1 and mTORC2 activity, respectively, which have been widely used for investigating functions of rapamycin and mTOR signaling (Lamming et al., 2012; Umemura et al., 2014).

To further illustrate rapamycin’s modulatory effect on CTGF, we stably knocked down the expression of mTOR, Raptor, or Rictor in LE/6 and WB-F344 cells using lentivirus carrying specific shRNAs(**Figures 2B–D**). We found that the expression of CTGF was greatly increased after knockdown of mTOR, Raptor, or Rictor (**Figures 2E–G**). Transcriptional response assay showed that the CTGF-luc activity was significantly increased after stable knockdown of mTOR (37.67 ± 3.58 vs 66.14 ± 2.71 in LE/6, *p* < 0.001; 22.67 ± 2.58 vs 42.22 ± 3.17 in WB-F344, *p* < 0.01) (**Figure 2H**). These results suggested that rapamycin upregulated CTGF expression through inactivation of mTOR signaling, and knockdown of mTOR, Raptor, or Rictor mimic the effect of rapamycin.

### Rapamycin Activates PI3K-Akt Signaling, Which in Turn Inhibits CTGF Expression in HPCs

**FIGURE 3:**
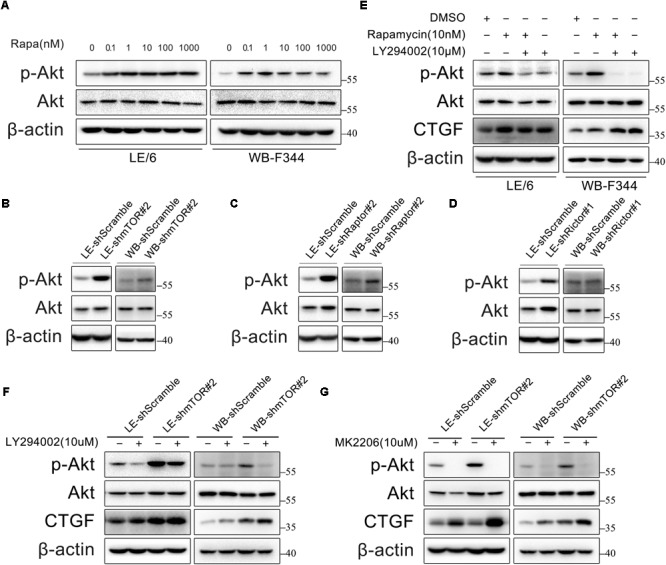
Rapamycin activates PI3K-Akt signaling, which in turn inhibits CTGF expression in HPCs. **(A)** LE/6 and WB-F344 cells were treated with rapamycin at indicated concentrations for 6 h. Lysates were subjected to Western blot analysis with antibodies against indicated proteins. β-actin was used as a loading control; **(B**–**D)** LE/6 and WB-F344 cells were transfected with lentivirus carrying shRNA against mTOR, Raptor, Rictor, or scramble shRNA and Western blot analysis showed the expression of these proteins. β-actin was used as a loading control; **(E)** LE/6 and WB-F344 cells were treated with rapamycin and LY294002 for 6 h. Lysates were subjected to Western blot analysis with antibodies against indicated proteins. β-actin was used as a loading control; **(F,G)** LE-shmTOR#2, WB-shmTOR#2 and their scramble control (shScramble) were incubated with LY294002 or MK2206 at indicated concentrations for 6 h. Lysates were subjected to Western blot analysis with antibodies against indicated proteins. β-actin was used as a loading control.

Previous studies have found that inhibition of mTOR leads to PI3K-Akt activation via a negative feedback loop originating from S6K1 (Wullschleger et al., 2006; Carracedo et al., 2008). Thus, we examined the phosphorylation of Akt in HPCs under rapamycin stimulation. As expected, rapamycin (0.1–1000 nM) activated PI3K-Akt signaling as indicated by phosphorylation of Akt (**Figure 3A**). In addition, we found that either knockdown of mTOR, Raptor, or Rictor alone resulted in Akt phosphorylation via negative feedback loop (**Figures 3B–D**). We then examined whether PI3K-Akt signaling is involved in the expression of CTGF in HPCs. Surprisingly, LY294002, an inhibitor of the PI3K-Akt pathway, failed to block basal and rapamycin-induced CTGF expression but promote the expression of CTGF (**Figure 3E**). Furthermore, inhibition of hyperactive PI3K-Akt pathway with LY294002 couldn’t block CTGF upregulation after knock down of mTOR (**Figure 3F**; **Supplementary Figure 2**). We used another specific Akt inhibitor, MK2206, to provide further evidence that Akt phosphorylation did not contribute to CTGF upregulation but inhibited CTGF expression (**Figure 3G**; **Supplementary Figure 2**). Taken together, we speculated that rapamycin promoted CTGF expression through other signaling pathways while PI3K-Akt activation via negative feedback loop limited the expression of CTGF to some extent.

### Rapamycin Activates the TGF-β-Smad Signaling

Transforming growth factor β is regarded as the master cytokine of liver fibrogenesis (Leask and Abraham, 2004; Inagaki and Okazaki, 2007). Previous studies have indicated that TGF-β was a major inducer of CTGF in liver fibrosis, and it upregulated CTGF expression in various cell types including HPCs (Wang et al., 2009; Ding et al., 2013). Meanwhile, CTGF is also an important downstream mediator of TGF-β and is vital for TGF-β induced liver fibrogenesis (Inagaki and Okazaki, 2007). On the other hand, rapamycin has been reported to activate TGF-β-Smad signaling in rat mesangial cells and prostate cancer cells (van der Poel, 2004; Osman et al., 2009). These findings strongly suggested that TGF-β-Smad signaling was involved in rapamycin induced CTGF expression in HPCs.

TGF-β-Smad signaling is initiated by the binding of TGF-β with TGF-β receptor type I (TGFβRI) and receptor type II (TGFβRII) serine/threonine kinases on the cell surface. This allows TGFβRII to phosphorylate the glycine-serine (GS) region of TGFβRI, which lead to TGFβRI activation. The activated TGFβRI recruits and activates the receptor-Smads (R-Smads) as indicated by C-terminal phosphorylation of Smad2 (Ser465/467) and Smad3 (Ser423/425). These activated R-Smads form heteromeric complexes with the Co-Smad (Smad4) and translocate into the nucleus to regulate the transcription of target genes (Shi and Massague, 2003; Massagué, 2012).

**FIGURE 4:**
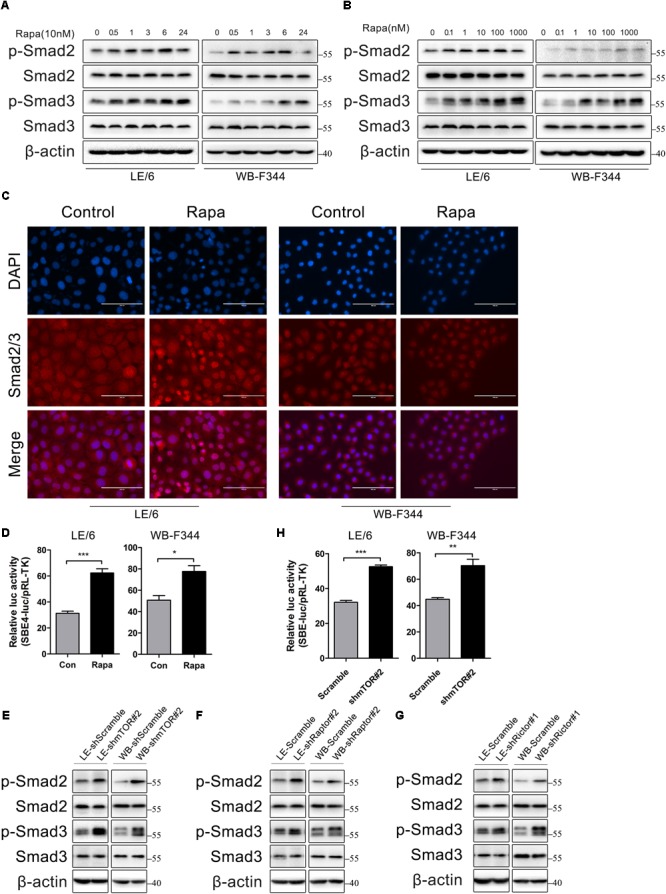
Rapamycin actives TGF-β-Smad signaling in HPCs. **(A)** LE/6 and WB-F344 cells were stimulated with rapamycin (10 nM) for the indicated times before cells were harvested for immunoblotting analysis against indicated proteins. β-actin was used as a loading control; **(B)** LE/6 and WB-F344 cells were treated with rapamycin at indicated concentrations for 6 h. Lysates were subjected to Western blot analysis with antibodies against indicated proteins. β-actin was used as a loading control; **(C)** LE/6 and WB-F344 cells were treated with rapamycin (10 nM) for 6 h and then subjected to immunofluorescent staining of Smad2/3 (red); DAPI were used to show the location of the nucleus (blue); scale bar, 100 μm; **(D)** LE/6 and WB-F344 cells were co-transfected with pRL-TK and CTGF-luc plasmids and then treated with Rapamycin for 16 h. Luciferase activity was normalized to renilla luciferase activity. Results showed as means ± SD. of triplicate measurements. ^∗^*p* < 0.05, ^∗∗∗^*p* < 0.001; **(E–G)** Lysates of LE-shmTOR#2, WB-shmTOR#2, LE-shRaptor#2, WB-shRaptor#2, LE-shRictor#1, WB-shRictor#1 and their scramble control (shScramble) were subjected to Western blot analysis with antibodies against indicated proteins. β-actin was used as a loading control; **(H)** LE-shmTOR#2, WB-shmTOR#2 and their scramble control (shScramble) were co-transfected with pRL-TK and SBE4-luc plasmids for 24 h. Luciferase activity was normalized to renilla luciferase activity. Results showed as means ± SD. of triplicate measurements. ^∗∗^*p* < 0.01, ^∗∗∗^*p* < 0.001.

So, we next stimulated HPCs with rapamycin and measured the phosphorylation state of Smad2 and Smad3 by western immunoblotting. We found that the phosphorylation levels of Smad2 and Smad3 were elevated after treated with rapamycin in a time and dose dependent manner (**Figures 4A,B**). Since the activated Smad complexes are translocated into the nucleus, we next monitored the nuclear import of these R-Smad proteins. Immunofluorescence showed that rapamycin induced Smad2 and Smad3 translocation into the nucleus (**Figure 4C**). Transcriptional response assay revealed that the Smad binding element luciferase (SBE4-luc) activity was significantly increased after rapamycin stimulation (31.28 ± 2.86 vs 62.33 ± 5.47 in LE/6, *p* < 0.001; 50.63 ± 7.50 vs 77.57 ± 9.37 in WB-F344, *p* < 0.05) (**Figure 4D**). These results demonstrated that TGF-β-Smad signaling was activated in response to rapamycin stimulation in HPCs.

As rapamycin exerts its function through inactivation of mTOR signaling and knockdown of mTOR, Raptor, or Rictor mimic the effect of rapamycin, so we next assessed whether knockdown of mTOR, Raptor, or Rictor could activate TGF-β-Smad signaling. Results showed that knockdown of mTOR, Raptor, or Rictor rendered phosphorylation of Smad2 and Smad3 upregulated (**Figures 4E–G**). The SBE4-luc activity was also upregulated after stable knockdown of mTOR (32.08 ± 1.51 vs 52.52 ± 1.78 in LE/6, *p* < 0.001; 44.79 ± 2.06 vs 70.26 ± 8.39 in WB-F344, *p* < 0.01) (**Figure 4H**). These results implied that inhibition of mTOR signaling upregulated TGF-β-Smad signaling in HPCs.

### TGFβ Receptor Is Involved in Rapamycin-Induced Upregulation of CTGF Expression

**FIGURE 5:**
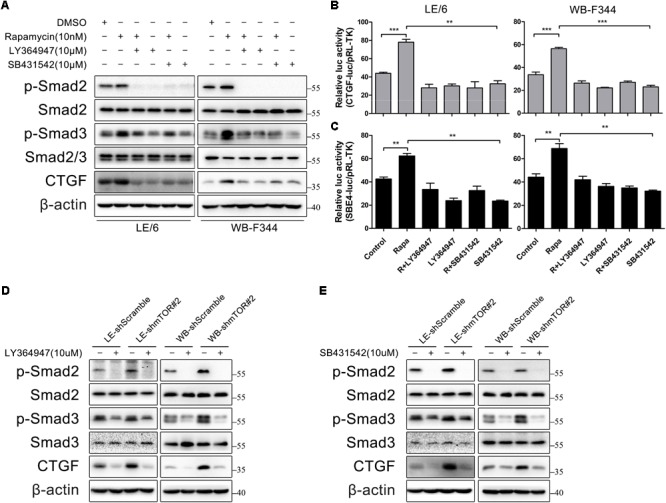
TGFβ receptor is involved in rapamycin-induced upregulation of CTGF expression. **(A)** LE/6 and WB-F344 cells were treated with rapamycin and indicated inhibitors for 6 h. Lysates were subjected to Western blot analysis with antibodies against indicated proteins. β-actin was used as a loading control; **(B,C)** LE/6 and WB-F344 cells were co-transfected with pRL-TK and CTGF-luc or SBE4-luc plasmids and then treated with rapamycin and indicated inhibitors for 16 h. Luciferase activity was normalized to renilla luciferase activity. Results showed as means ± SD of triplicate measurements. ^∗∗^*p* < 0.01, ^∗∗∗^*p* < 0.001. R, rapamycin; **(D,E)** LE-shmTOR#2, WB-shmTOR#2 and their scramble control (shScramble) were incubated with LY364947 or SB431542 at indicated concentrations for 6 h. Lysates were subjected to Western blot analysis with antibodies against indicated proteins. β-actin was used as a loading control.

Next, we focused on the contributions of TGF-β-Smad signaling which was activated after stimulated with rapamycin. We used two TGF-β-Smad signaling inhibitors, LY364947 and SB431542, both of which were TGFβRI serine/threonine kinase (ALK5) inhibitors. As shown in **Figure 5A**, after LE/6 and WB-F344 cells were treated with these two kinase inhibitors, both basal and rapamycin induced phosphorylation of Smad2 were reduced to nearly undetectable levels, while phosphorylation of Smad3 was partially impaired. Further, basal and rapamycin induced CTGF expression were completely blocked. Transcriptional response assay revealed that both CTGF-luc and SBE4-luc activities were reduced after incubation with LY364947 or SB431542 (**Figures 5B,C**). Similarly, the phosphorylation of Smad2 was reduced below basal levels, phosphorylation of Smad3 was reduced, and expression of CTGF was completely blocked in LE-shmTOR#2 and WB- shmTOR#2 cells treated with these ALK5 kinase inhibitors (**Figures 5D,E, Supplementary Figure 2A**). Collectively, these results revealed that TGFβ receptor was involved in rapamycin-induced upregulation of CTGF expression.

Besides the canonical Smad signaling, Smad-independent signaling such as the MAPK signaling (MEK1/2, JNK, p38MAPK) have also been implicated in TGFβ signaling (Derynck and Zhang, 2003; Zhang, 2009). To test whether these Smad-independent signaling were involved in rapamycin induced CTGF expression, we treated LE/6shmTOR#2 cells with various MAPK inhibitors. As shown in **Supplementary Figure 2A**, SP600125 (JNK inhibitor), U0126 (MEK1/2 inhibitor suppressed Erk signaling), and SB203580 (p38MAPK inhibitor) had no effect on CTGF expression. These results suggested that MAPK signaling was not involved in rapamycin-induced upregulation of CTGF expression.

### Smad2, but Not Smad3, Is Involved in CTGF Induction Through Rapamycin-Activated TGF-β-Smad Signaling

**FIGURE 6:**
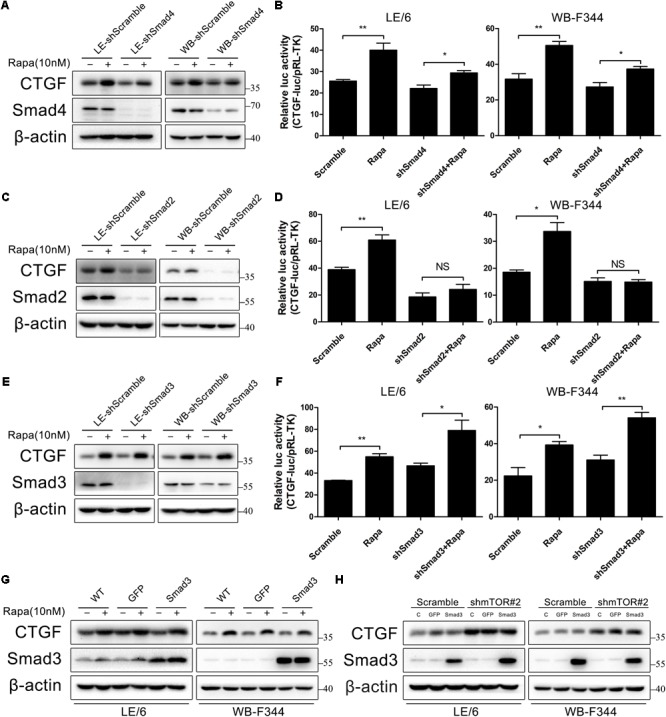
Smad2, but not smad3, is involved in CTGF induction through rapamycin-activated TGF-β-Smad signaling. **(A–E)** LE-shSmad4, WB-shSamd4, LE-shSmad2, WB-shSamd2, LE-shSmad3, WB-shSamd3 and their scramble control (shScramble) were treated with rapamycin (10 nM) for 6 h. Lysates were subjected to Western blot analysis with antibodies against indicated proteins. β-actin was used as a loading control; **(B,D,F)** LE-shSmad4, WB-shSamd4, LE-shSmad2, WB-shSamd2, LE-shSmad3, WB-shSamd3 and their scramble control (shScramble) were co-transfected with pRL-TK and SBE4-luc plasmids for 24 h and then treated with rapamycin (10 nM) for 16 h. Luciferase activity was normalized to renilla luciferase activity. Results showed as means ± SD of triplicate measurements. ^∗^*p* < 0.05, ^∗∗^*p* < 0.01, NS, no significance; **(G)** LE/6 and WB-F344 cells were infected with Ad-GFP and Ad-Smad3 for 72 h. Then, cells were treated with rapamycin (10 nM) for 6 h. Lysates were subjected to Western blot analysis with antibodies against CTGF and Smad3. β-actin was used as a loading control; **(H)** LE-shmTOR#2, WB-shmTOR#2 and their scramble control (shScramble) were infected with Ad-GFP and Ad-Smad3 for 72 h before cells were harvested for Western blot analysis with antibodies against CTGF and Smad3.

Generally speaking, the canonical TGF-β signaling is propagated through the way that activated Smad2 and Smad3 heterodimerize with Smad4 to build up a transcriptionally active complex, and translocate into the nucleus to modulate target gene expression. We next investigated the role of these Smad proteins in rapamycin-induced CTGF upregulation using lentivirus to stably knock down the expression of Smad4, Smad2, and Smad3. As shown in **Figures 6A,B**, knockdown of Smad4 partially blocked CTGF protein expression and CTGF-luc activity in response to rapamycin (LE-shSmad4 vs LE-shSmad4+Rapa: 22.09 ± 2.84 vs 29.39 ± 1.88, *p* < 0.05; WB-shSmad4 vs WB-shSmad4+Rapa: 27.33 ± 4.24 vs 37.33 ± 2.50, *p* < 0.05). Besides, knockdown of Smad2 strongly reduced CTGF protein expression in the presence of rapamycin (**Figure 6C**). Transcriptional response assay showed that the CTGF-luc activity was not responsive to rapamycin after knockdown of Smad2 (LE-shSmad2 vs LE-shSmad2+Rapa: 18.57 ± 5.20 vs 24.19 ± 6.56, *p* > 0.05; WB-shSmad2 vs WB-shSmad2+Rapa: 15.10 ± 2.32 vs 14.88 ± 1.55, *p* > 0.05) (**Figure 6D**). To our surprise, knockdown of Smad3 had no effect on rapamycin-induced CTGF expression in HPCs (**Figure 6E**), and CTGF-luc activity was still upregulated by rapamycin after stable knockdown of Smad3 (LE-shSmad3 vs LE-shSmad3+Rapa: 46.68 ± 4.29 vs 79.01 ± 16.39, *p* < 0.05; WB-shSmad2 vs WB-shSmad2+Rapa: 31.06 ± 4.59 vs 54.12 ± 5.07, *p* < 0.01) (**Figure 6F**). To further investigate the role of Smad3 in rapamycin-induced upregulation of CTGF, we used an adenovirus to overexpress Smad3 in HPCs. Consistent with knockdown studies, Smad3 overexpression had no effect on rapamycin-induced CTGF expression (**Figure 6G**). Moreover, Smad3 overexpression after knockdown of mTOR did not alter the expression of CTGF (**Figure 6H**). The results presented above suggested that Smad2 and to some extent Smad4, but not smad3, were involved in the induction of CTGF through rapamycin-activated TGF-β-Smad signaling.

### Rapamycin Induces ROS Generation and Latent TGF-β Activation, Which Contribute to TGF-β-Smad Signaling

Transforming growth factor-β is produced in a latent complex and extracellular or intracellular activated by shedding the latency-associated protein (LAP), thereby converting the latent form into the active ligand capable of receptor binding (Khalil, 1999; Breitkopf, 2001). Our previous studies revealed that TGF-β was secreted in a latent form by HPCs and that latent TGF-β was autonomously and intracellularly activated to trigger Smad signaling (Ding et al., 2013). Both rapamycin and CNIs have been reported to activate latent TGF-β via a mechanism depend on ROS generation (Akool et al., 2008; Osman et al., 2009), so we investigated whether rapamycin-induced TGF-β-Smad activation depended on ROS in HPCs.

**FIGURE 7:**
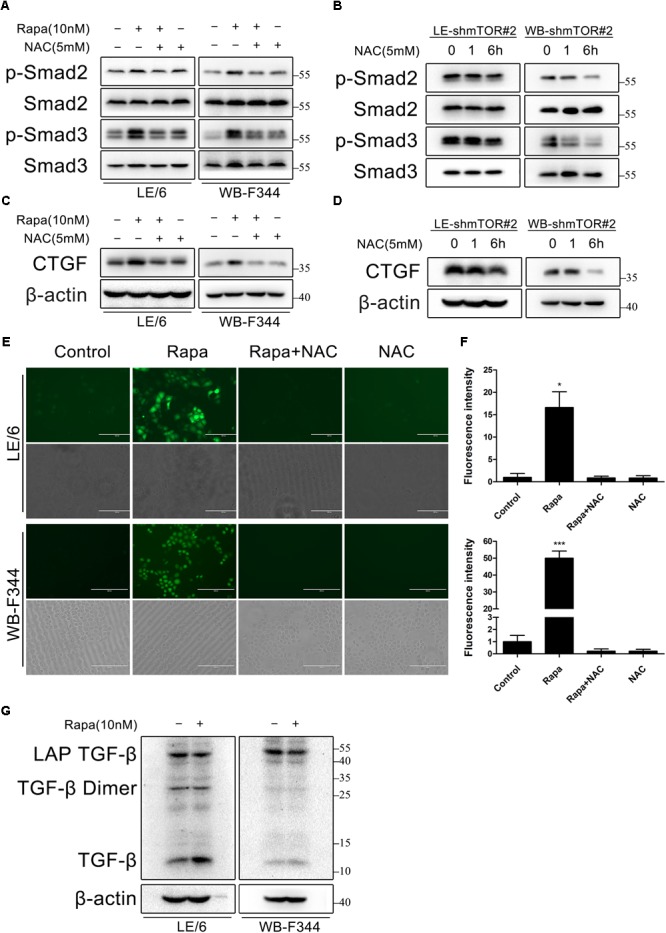
Rapamycin induces ROS generation and latent TGF-β activation, which contribute to TGF-β-Smad signaling. **(A,C)** LE/6 and WB-F344 cells were treated with rapamycin (10 nM) in the absence or presence of NAC (5 mM) for 6 h. Lysates were subjected to Western blot analysis with antibodies against indicated proteins. β-actin was used as a loading control; **(B,D)** LE-shmTOR#2 and WB-shmTOR#2 cells were incubated with NAC (5 mM) for 6 h. Lysates were subjected to Western blot analysis with antibodies against indicated proteins. β-actin was used as a loading control; **(E)** LE/6 and WB-F344 cells were treated with rapamycin (10 nM) in the absence or presence of NAC (5 mM) for 1 h. Then, cells were washed with PBS and incubated in the dark at 37°C for 30 min in 10 μM DCFH-DA. After washed with FBS free media three times, ROS generation was visualized by dichlorofluorescein (DCF) formation in inverse fluorescence microscopy. Corresponding phase contrast image was placed below. scale bar, 200 μm; **(F)** Quantification of dichlorofluorescein (DCF) formation intensity using IPP software. Data represented means ± SD. (*n* = 3). ^∗^*p* < 0.05 compared with other groups; **(G)** LE/6 and WB-F344 cells were treated with 10 nM rapamycin for 6 h. Lysates were subjected to Western blot analysis with antibodies against TGF-β. β-actin was used as a loading control.

To this end, we treated LE/6 and WB-F344 cells with rapamycin (10 nM) in the absence or presence of the ROS scavenger *N*-acetyl-L-cysteine (NAC, 5 mM). As shown in **Figure 7A**, phosphorylation of Smad2 and Smad3 induced by rapamycin was almost completely inhibited by NAC. NAC also decreased phosphorylation of Smad2 and Smad3 after knock down of mTOR (**Figure 7B**). Simultaneously, Upregulation of CTGF by either rapamycin treatment or mTOR knockdown was also almost completely blocked by NAC treatment (**Figures 7C,D**). We next examined whether rapamycin increases ROS formation in HPCs. We used a fluorimetric probe named 2′,7′-DCFH-DA which is commonly used to detect ROS generation and the overall oxidative stress. Results showed that rapamycin caused a significant increase in ROS formation (**Figures 7E,F**). Furthermore, incubation with NAC abolished the rapamycin-triggered ROS production completely (**Figures 7E,F**). To confirm the intracellular activation of latent TGF-β by rapamycin, we performed western blotting to detect latent TGF-β and active TGF-β. Results showed that rapamycin treatment decreased latent TGF-β and increased active TGF-β in HPCs (**Figure 7G**). Collectively, these results demonstrated that rapamycin-induced ROS formation contributed to the activation of latent TGF-β, which can then bind to the TGF-β receptor and activate TGF-β-Smad signaling, leading to induction of CTGF expression.

## Discussion

**FIGURE 8:**
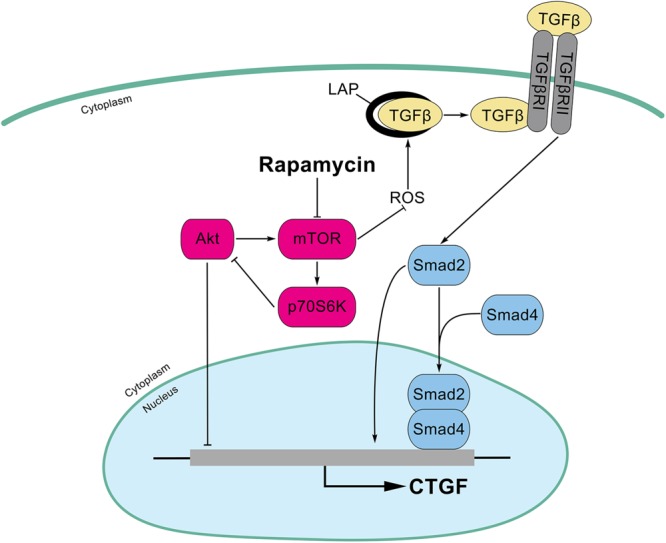
Schematic illustrations of this study. Inhibition of mTOR activity with rapamycin results in a hyperactive PI3K-Akt pathway, whereas this activation inhibits the expression of CTGF in HPCs. In addition, rapamycin increased ROS generation and latent TGF-β activation which subsequently activated TGF-β-Smad2 signaling to promote CTGF expression.

In this study, we found that rapamycin at nanomolar concentration upregulated CTGF expression at the transcriptional level in HPCs. Mechanistically, inhibition of mTOR activity with rapamycin resulted in a hyperactive PI3K-Akt pathway, whereas this activation inhibited the expression of CTGF in HPCs. Besides, rapamycin increased ROS generation and subsequently activated TGF-β-Smad2 signaling to promote CTGF expression (**Figure 8**). Expansion of HPCs occurs post liver transplantation and participates in the pathophysiologic changes of grafts in recipients. First, HPCs response are increased and implicated in the progression of fibrosis associated with hepatitis C recurrence after liver transplantation (Prakoso et al., 2014; Sclair et al., 2016). Second, small-for-size partial liver transplantation such as split liver transplantation, living donor graft and reduced-size graft increase the availability of livers and partially overcome the shortage of organs. It has been reported that HPCs are activated after small-for-size liver transplantation in rats where the small liver graft needed to regenerate (Mao et al., 2008). Third, hepatocyte replication is impaired in steatotic liver regeneration after living donor transplantation, and expansion of HPCs compensates for impaired hepatocyte replication (Cho et al., 2010). Taken together, we speculated that rapamycin, a commonly used antirejection agent after liver transplantation, may have potential fibrotic effect through activating profibrotic TGF-β-Smad signaling and upregulating profibrotic factor CTGF expression in HPCs.

Connective tissue growth factor is strongly upregulated in fibrotic liver tissue and plays a pivotal role in fibrogenesis of liver (Gressner and Gressner, 2008; Weiskirchen, 2016). It is synthesized and released by various cell types in liver including hepatocytes, cholangiocytes, HSCs, and HPCs (Gressner et al., 2007; Ding et al., 2013, 2016; Williams et al., 2014). Besides, as a downstream modulator protein of TGF-β, CTGF is thought to amplify the profibrogenic action of TGF-β (Leask and Abraham, 2006; Gressner et al., 2009). In this study, we found that rapamycin upregulated CTGF expression at the transcriptional level in HPCs but not in hepatocytes and hepatoma cells. Previous studies have demonstrated that rapamycin showed controversial roles in renal and lung fibrosis duo to its different roles in different cells. Most studies in liver have shown that rapamycin represented an anti-fibrosis role (Zhu et al., 1999; Biecker et al., 2005; Neef et al., 2006), while our study found that rapamycin upregulated the profibrotic factor CTGF expression in HPCs. Presumably, the overall effect of rapamycin on liver fibrosis is determined by the balance of the controversial functions on various structural cells in liver. In most previous studies, HPCs were only slightly expanded under the experimental or clinical conditions, so the overall effect of rapamycin was anti-fibrotic. Further investigation in future studies are needed to unravel rapamycin’s effect on liver fibrosis in a vivo model which HPCs expanded largely.

Rapamycin exerts its effects mainly through inhibition of mTOR signaling. In this study, as well, we found that rapamycin upregulated CTGF by blocking mTOR signaling. Knockdown of mTOR, Raptor, or Rictor significantly upregulated CTGF expression as expected, which indicated that both mTORC1 and mTORC2 were involved in rapamycin’s effects on CTGF expression. But how does rapamycin, through inhibiting mTOR signaling, promote CTGF expression of HPCs? We observed that both rapamycin and mTOR knockdown caused PI3K-Akt activation which may through a negative feedback loop originating from S6K1 as reported by other groups (Wullschleger et al., 2006; Carracedo et al., 2008). Previous studies showed that rapamycin promoted CTGF expression of lung fibroblasts and epithelial cells via PI3K-Akt (Xu et al., 2013, 2015). However, our studies showed that inhibiting feedback activated PI3K-Akt with LY294002 or MK2206 did not block CTGF expression induced by mTOR inhibition. In the contrast, PI3K-Akt activation via the negative feedback loop limited the expression of CTGF after rapamycin treatment to some extent, at odds with previous studies showing that Akt signaling promoted CTGF expression in other cell types (Xu et al., 2013, 2015). The molecular mechanisms through which PI3K/Akt signaling inhibits CTGF expression warrants further study.

In a further attempt to reveal the underlying mechanisms for rapamycin-induced CTGF upregulation, we found an activation of TGF-β-Smad signaling. TGF-β receptor kinase inhibitors completely blocked rapamycin and mTOR knockdown induced CTGF upregulation. Our results suggested that TGF-β-Smad signaling was indispensable for rapamycin-induced expression of CTGF. Other findings are consistent with ours. Osman et al. (2009) reported that rapamycin induced rapid activation of TGF-β-Smad signaling in rat mesangial cells. Another study also reported that rapamycin further augmented SBE4-luc activation in prostate cancer cells (van der Poel, 2004). Shihab et al. (2004) found that rapamycin increased TGF-β expression in a rat model. Moreover, rapamycin can endow constitutive TGFβ signaling in monkey kidney COS-1 cells through binding to its intracellular receptor FKBP12, which inhibits TGFβ type I receptor phosphorylation (Chen et al., 1997). Together with our study, we suggested that inhibition of mTOR signaling by rapamycin may secondarily activate TGF-β-Smad signaling.

Distinct roles of Smad2 and Smad3 in TGF-β-Smad signaling have been reported by many studies (Kretschmer et al., 2003; Brown et al., 2007; Weng et al., 2007; Gressner et al., 2009). Even in hepatocyte, different roles of Smad2 and Smad3 have been reported. Gressner et al. (2009) reported selective transcriptional activation of the CTGF promoter by Smad2 (but not Smad3) in hepatocytes isolated from male Sprague-Dawley rats, while Weng et al. (2007) found that TGF-β-induced CTGF expression was mediated by the ALK5-Smad3 pathway in hepatocytes isolated from livers of male C57/BL-6 mice. This seems rather paradoxical. The possible reason for those contrary results is that they isolate hepatocytes from different species, which suggested that these Smads have distinct functions in TGF-β-induced CTGF expression across species. In accord with Kretschmer, who found that Smad2 and Smad3 have distinct roles in different cell types (Kretschmer et al., 2003), we suggested that TGF-β-Smad signaling induced CTGF expression is highly species and cell specific. In the present study, knockdown of Smad2 was sufficient to almost completely reverse rapamycin-induced CTGF upregulation and knockdown of Smad4 partially blocked CTGF upregulation, whereas knockdown of Smad3 had no effect. This finding is in consistent with Gressner’s study, both of which are conducted in rat. Based on the differential responses to rapamycin in Smad2 and Smad3 knockdown cells in our study, we suggest a predominant role of Smad2 protein in the transcriptional activation of the CTGF promoter in HPCs.

TGF-β is regarded as the master cytokine of liver fibrogenesis (Leask and Abraham, 2004; Inagaki and Okazaki, 2007). The biological activity of TGF-β is restrained by secretion as a latent complex in which TGF-beta homodimers are non-covalently associated with homodimers of their respective pro-peptide called the LAP (Khalil, 1999; Breitkopf, 2001; Jobling et al., 2006). Release of TGF-β from the latent complex, which is referred to as activation, allows TGF-β to bind its cellular receptors. ROS generation has been implicated in this process. Specifically, ROS-induced oxidation at specific amino acids of LAP triggers a conformational change allowing rapid release of TGF-β (Barcellos-Hoff and Dix, 1996; Jobling et al., 2006). A previous study demonstrated that rapamycin activates latent TGF-β via a mechanism depend on ROS generation (Osman et al., 2009). Ours results are consistent with those reports, we found that rapamycin-induced ROS formation contributed to the activation of latent TGF-β thus activating TGF-β-Smad signaling. The mechanism by which rapamycin triggers intracellular ROS formation in HPCs still needs further investigation.

## Conclusion

In conclusion, we demonstrate that rapamycin at nanomolar concentrations upregulates CTGF expression in HPCs. Inhibition of mTOR activity with rapamycin results in a hyperactive PI3K-Akt pathway, but this actually inhibits the expression of CTGF in HPCs. In addition, Smad2, but not smad3, is involved in the induction of CTGF through rapamycin-activated TGF-β-Smad signaling. Furthermore, we find that rapamycin induces ROS generation and latent TGF-β activation which contribute to TGF-β-Smad signaling (**Figure 8**). Considering that the HPCs are expanded under specific circumstance after liver transplantation, we speculate that HPCs may an important source of CTGF during rapamycin anti-rejection treatment, and this effect may potentiate fibrosis of liver grafts. Together with many unexpected outcomes about rapamycin in liver transplantation (Kawahara et al., 2011; Massoud and Wiesner, 2012), it is necessary to further investigate the molecular mechanism of rapamycin.

## Author Contributions

YW performed the experiments, designed the figures, and drafted the manuscript. YW, WW, and Z-yD designed the research and analyzed the data. WW, X-mP, YH, and Y-xX were also involved in performing experiments. H-fL, LC, B-xZ, and X-pC assisted with analyzing and interpreting data and provided technical support. Z-yD and X-pC critical revised manuscript for important intellectual content and obtained funding. All authors read and approved the final manuscript.

## Conflict of Interest Statement

The authors declare that the research was conducted in the absence of any commercial or financial relationships that could be construed as a potential conflict of interest.

## Abbreviations

4E-BP1: eukaryotic initiation factor 4E binding protein 1
BDL: bile duct ligation
CNIs: calcineurin inhibitors
CTGF: connective tissue growth factor
DCF: 2′,7′-dichlorofluorescein
DCFH: 2′,7′-dichlorodihydrofluorescein
DCFH-DA: 2′,7′-Dichlorodihydrofluorescein diacetate
FKBP12: FK506-binding protein of 12 kDa
HCC: hepatocellular carcinoma
HPCs: hepatic progenitor cells
HRP: Horseradish peroxidase
HSCs: hepatic stellate cells
LAP: latency associated protein
MAPK: mitogen-activated protein kinase
mTOR: mechanistic target of rapamycin
NAC: *N*-acetyl-L-cysteine
PAI-1: plasminogen activator inhibitor 1
Raptor: regulatory protein associated with mTOR
ROS: reactive oxygen species
Rictor: rapamycin insensitive companion of mTOR
SBE: Smad binding element
shRNA: small hairpin RNA
TGF-β: transforming growth factor β
TGFβRI: TGF-β receptor type I
TGFβRII: TGF-β receptor type II

## References

[B1] Akool E. Doller A. Babelova A. Tsalastra W. Moreth K. Schaefer L. (2008). Molecular mechanisms of TGF beta receptor-triggered signaling cascades rapidly induced by the calcineurin inhibitors cyclosporin A and FK506. *J. Immunol.* 181 2831–2845. 10.4049/jimmunol.181.4.2831 18684975

[B2] Asrani S. K. Leise M. D. West C. P. Murad M. H. Pedersen R. A. Erwin P. J. (2010). Use of sirolimus in liver transplant recipients with renal insufficiency: a systematic review and meta-analysis. *Hepatology* 52 1360–1370. 10.1002/hep.23835 20815021 PMC4130484

[B3] Barcellos-Hoff M. H. Dix T. A. (1996). Redox-mediated activation of latent transforming growth factor-beta 1. *Mol. Endocrinol.* 10 1077–1083. 10.1210/mend.10.9.8885242 8885242

[B4] Bataller R. Brenner D. A. (2005). Liver fibrosis. *J. Clin. Invest.* 115 209–218. 10.1172/JCI200524282 15690074 PMC546435

[B5] Biecker E. De Gottardi A. Neef M. Unternahrer M. Schneider V. Ledermann M. (2005). Long-term treatment of bile duct-ligated rats with rapamycin (sirolimus) significantly attenuates liver fibrosis: analysis of the underlying mechanisms. *J. Pharmacol. Exp. Ther.* 313 952–961. 10.1124/jpet.104.079616 15769867

[B6] Braun L. Goyette M. Yaswen P. Thompson N. L. Fausto N. (1987). Growth in culture and tumorigenicity after transfection with the ras oncogene of liver epithelial cells from carcinogen-treated rats. *Cancer Res.* 47 4116–4124. 2440558

[B7] Breitkopf K. (2001). Expression and matrix deposition of latent transforming growth factor β binding proteins in normal and fibrotic rat liver and transdifferentiating hepatic stellate cells in culture. *Hepatology* 33 387–396. 10.1053/jhep.2001.21996 11172340

[B8] Brown K. A. Pietenpol J. A. Moses H. L. (2007). A tale of two proteins: differential roles and regulation of Smad2 and Smad3 in TGF-beta signaling. *J. Cell. Biochem.* 101 9–33. 10.1002/jcb.21255 17340614

[B9] Carracedo A. Ma L. Teruya-Feldstein J. Rojo F. Salmena L. Alimonti A. (2008). Inhibition of mTORC1 leads to MAPK pathway activation through a PI3K-dependent feedback loop in human cancer. *J. Clin. Invest.* 118 3065–3074. 10.1172/JCI34739 18725988 PMC2518073

[B10] Chen Y. G. Liu F. Massague J. (1997). Mechanism of TGFbeta receptor inhibition by FKBP12. *EMBO J.* 16 3866–3876. 10.1093/emboj/16.13.3866 9233797 PMC1170011

[B11] Cho J. Y. Suh K. S. Shin W. Y. Lee H. W. Yi N. J. Kim M. A. (2010). Expansion of hepatic progenitor cell in fatty liver graft after living donor liver transplantation. *Transpl. Int.* 23 530–537. 10.1111/j.1432-2277.2009.01020.x 20003044

[B12] Clouston A. D. Powell E. E. Walsh M. J. Richardson M. M. Demetris A. J. Jonsson J. R. (2005). Fibrosis correlates with a ductular reaction in hepatitis C: roles of impaired replication, progenitor cells and steatosis. *Hepatology* 41 809–818. 10.1002/hep.20650 15793848

[B13] Derynck R. Zhang Y. E. (2003). Smad-dependent and Smad-independent pathways in TGF-beta family signalling. *Nature* 425 577–584. 10.1038/nature02006 14534577

[B14] Ding Z. Jin G. Liang H. Wang W. Chen W. Datta P. K. (2013). Transforming growth factor β induces expression of connective tissue growth factor in hepatic progenitor cells through Smad independent signaling. *Cell. Signal.* 25 1981–1992. 10.1016/j.cellsig.2013.05.027 23727026

[B15] Ding Z. Jin G. Wang W. Sun Y. Chen W. Chen L. (2016). Activin A-smad signaling mediates connective tissue growth factor synthesis in liver progenitor cells. *Int. J. Mol. Sci.* 17 408. 10.3390/ijms17030408 27011166 PMC4813263

[B16] Dull T. Zufferey R. Kelly M. Mandel R. J. Nguyen M. Trono D. (1998). A third-generation lentivirus vector with a conditional packaging system. *J. Virol.* 72 8463–8471. 9765382 10.1128/jvi.72.11.8463-8471.1998 PMC110254

[B17] Duncan A. W. Dorrell C. Grompe M. (2009). Stem cells and liver regeneration. *Gastroenterology* 137 466–481. 10.1053/j.gastro.2009.05.044 19470389 PMC3136245

[B18] Fattovich G. Stroffolini T. Zagni I. Donato F. (2004). Hepatocellular carcinoma in cirrhosis: incidence and risk factors. *Gastroenterology* 127 S35–S50. 10.1053/j.gast.2004.09.014 15508101

[B19] Gao Y. Xu X. Ding K. Liang Y. Jiang D. Dai H. (2013). Rapamycin inhibits transforming growth factor β1-induced fibrogenesis in primary human lung fibroblasts. *Yonsei Med. J.* 54 437–444. 10.3349/ymj.2013.54.2.437 23364979 PMC3576000

[B20] Gressner O. A. Gressner A. M. (2008). Connective tissue growth factor: a fibrogenic master switch in fibrotic liver diseases. *Liver Int.* 28 1065–1079. 10.1111/j.1478-3231.2008.01826.x 18783549

[B21] Gressner O. A. Lahme B. Demirci I. Gressner A. M. Weiskirchen R. (2007). Differential effects of TGF-β on connective tissue growth factor (CTGF/CCN2) expression in hepatic stellate cells and hepatocytes. *J. Hepatol.* 47 699–710. 10.1016/j.jhep.2007.05.015 17629588

[B22] Gressner O. A. Lahme B. Siluschek M. Rehbein K. Weiskirchen R. Gressner A. M. (2009). Connective tissue growth factor is a Smad2 regulated amplifier of transforming growth factor β actions in hepatocytes-But without modulating bone morphogenetic protein 7 signaling. *Hepatology* 49 2021–2030. 10.1002/hep.22850 19309720

[B23] Inagaki Y. Okazaki I. (2007). Emerging insights into transforming growth factor beta Smad signal in hepatic fibrogenesis. *Gut* 56 284–292. 10.1136/gut.2005.088690 17303605 PMC1856752

[B24] Jobling M. F. Mott J. D. Finnegan M. T. Jurukovski V. Erickson A. C. Walian P. J. (2006). Isoform-specific activation of latent transforming growth factor beta (LTGF-beta) by reactive oxygen species. *Radiat. Res.* 166 839–848. 10.1667/RR0695.1 17149983

[B25] Kawahara T. Asthana S. Kneteman N. M. (2011). m-TOR inhibitors: what role in liver transplantation? *J. Hepatol.* 55 1441–1451. 10.1016/j.jhep.2011.06.015 21781947

[B26] Khalil N. (1999). TGF-beta: from latent to active. *Microbes Infect.* 1 1255–1263. 10611753 10.1016/s1286-4579(99)00259-2

[B27] Korfhagen T. R. Le Cras T. D. Davidson C. R. Schmidt S. M. Ikegami M. Whitsett J. A. (2009). Rapamycin prevents transforming growth factor-α–induced pulmonary fibrosis. *Am. J. Respir. Cell Mol. Biol.* 41 562–572. 10.1165/rcmb.2008-0377OC 19244201 PMC2778163

[B28] Kretschmer A. Moepert K. Dames S. Sternberger M. Kaufmann J. Klippel A. (2003). Differential regulation of TGF-beta signaling through Smad2, Smad3 and Smad4. *Oncogene* 22 6748–6763. 10.1038/sj.onc.1206791 14555988

[B29] Lamming D. W. Ye L. Katajisto P. Goncalves M. D. Saitoh M. Stevens D. M. (2012). Rapamycin-induced insulin resistance is mediated by mTORC2 loss and uncoupled from longevity. *Science* 335 1638–1643. 10.1126/science.1215135 22461615 PMC3324089

[B30] Leask A. Abraham D. J. (2004). TGF-beta signaling and the fibrotic response. *FASEB J.* 18 816–827. 10.1096/fj.03-1273rev 15117886

[B31] Leask A. Abraham D. J. (2006). All in the CCN family: essential matricellular signaling modulators emerge from the bunker. *J. Cell Sci.* 119 4803–4810. 10.1242/jcs.03270 17130294

[B32] Lowes K. N. Brennan B. A. Yeoh G. C. Olynyk J. K. (1999). Oval cell numbers in human chronic liver diseases are directly related to disease severity. *Am. J. Pathol.* 154 537–541. 10.1016/S0002-9440(10)65299-6 10027411 PMC1849988

[B33] Mao L. Qiu Y. D. Fang S. Wu Y. F. Liu H. Ding Y. T. (2008). Liver progenitor cells activated after 30% small-for-size liver transplantation in rats: a preliminary study. *Transplant. Proc.* 40 1635–1640. 10.1016/j.transproceed.2008.03.133 18589164

[B34] Masola V. Carraro A. Zaza G. Bellin G. Montin U. Violi P. (2015). Epithelial to mesenchymal transition in the liver field: the double face of Everolimus in vitro. *BMC Gastroenterol.* 15:118. 10.1186/s12876-015-0347-r6 26369804 PMC4570634

[B35] Masola V. Zaza G. Granata S. Gambaro G. Onisto M. Lupo A. (2013). Everolimus-induced epithelial to mesenchymal transition in immortalized human renal proximal tubular epithelial cells: key role of heparanase. *J. Transl. Med.* 11:292. 10.1186/1479-5876-11-292 24256696 PMC4222256

[B36] Massagué J. (2012). TGFβ signalling in context. *Nat. Rev. Mol. Cell Biol.* 13 616–630. 10.1038/nrm3434 22992590 PMC4027049

[B37] Massoud O. Wiesner R. H. (2012). The use of sirolimus should be restricted in liver transplantation. *J. Hepatol.* 56 288–290. 10.1016/j.jhep.2011.06.012 21741926

[B38] Miller J. L. (1999). Sirolimus approved with renal transplant indication. *Am. J. Health Syst. Pharm.* 56 2177–2178. 10.1093/ajhp/56.21.2177 10565691

[B39] Moffat J. Grueneberg D. A. Yang X. Kim S. Y. Kloepfer A. M. Hinkle G. (2006). A lentiviral RNAi library for human and mouse genes applied to an arrayed viral high-content screen. *Cell* 124 1283–1298. 10.1016/j.cell.2006.01.040 16564017

[B40] Neef M. Ledermann M. Saegesser H. Schneider V. Reichen J. (2006). Low-dose oral rapamycin treatment reduces fibrogenesis, improves liver function, and prolongs survival in rats with established liver cirrhosis. *J. Hepatol.* 45 786–796. 10.1016/j.jhep.2006.07.030 17050028

[B41] Nguyen L. N. Furuya M. H. Wolfraim L. A. Nguyen A. P. Holdren M. S. Campbell J. S. (2007). Transforming growth factor-beta differentially regulates oval cell and hepatocyte proliferation. *Hepatology* 45 31–41. 10.1002/hep.21466 17187411

[B42] Osman B. Doller A. Akool E. Holdener M. Hintermann E. Pfeilschifter J. (2009). Rapamycin induces the TGFβ1/Smad signaling cascade in renal mesangial cells upstream of mTOR. *Cell. Signal.* 21 1806–1817. 10.1016/j.cellsig.2009.07.016 19666112

[B43] Patsenker E. Schneider V. Ledermann M. Saegesser H. Dorn C. Hellerbrand C. (2011). Potent antifibrotic activity of mTOR inhibitors sirolimus and everolimus but not of cyclosporine A and tacrolimus in experimental liver fibrosis. *J. Hepatol.* 55 388–398. 10.1016/j.jhep.2010.10.044 21168455

[B44] Pellicoro A. Ramachandran P. Iredale J. P. Fallowfield J. A. (2014). Liver fibrosis and repair: immune regulation of wound healing in a solid organ. *Nat. Rev. Immunol.* 14 181–194. 10.1038/nri3623 24566915

[B45] Pontrelli P. Rossini M. Infante B. Stallone G. Schena A. Loverre A. (2008). Rapamycin inhibits PAI-1 expression and reduces interstitial fibrosis and glomerulosclerosis in chronic allograft nephropathy. *Transplantation* 85 125–134. 10.1097/01.tp.0000296831.91303.9a 18192922

[B46] Prakoso E. Tirnitz-Parker J. E. Clouston A. D. Kayali Z. Lee A. Gan E. K. (2014). Analysis of the intrahepatic ductular reaction and progenitor cell responses in hepatitis C virus recurrence after liver transplantation. *Liver Transpl.* 20 1508–1519. 10.1002/lt.24007 25241637

[B47] Renken C. Fischer D. C. Kundt G. Gretz N. Haffner D. (2011). Inhibition of mTOR with sirolimus does not attenuate progression of liver and kidney disease in PCK rats. *Nephrol. Dial. Transplant.* 26 92–100. 10.1093/ndt/gfq384 20615907

[B48] Saxton R. A. Sabatini D. M. (2017). mTOR signaling in growth, metabolism, and disease. *Cell* 168 960–976. 10.1016/j.cell.2017.02.004 28283069 PMC5394987

[B49] Sclair S. N. Fiel M. I. Wu H. S. Doucette J. Aloman C. Schiano T. D. (2016). Increased hepatic progenitor cell response and ductular reaction in patients with severe recurrent HCV post-liver transplantation. *Clin. Transplant.* 30 722–730. 10.1111/ctr.12740 27027987

[B50] Servais A. Meas-Yedid V. Toupance O. Lebranchu Y. Thierry A. Moulin B. (2009). Interstitial fibrosis quantification in renal transplant recipients randomized to continue cyclosporine or convert to sirolimus. *Am. J. Transplant.* 9 2552–2560. 10.1111/j.1600-6143.2009.02803.x 19843033

[B51] Shamji A. F. Nghiem P. Schreiber S. L. (2003). Integration of growth factor and nutrient signaling: implications for cancer biology. *Mol. Cell* 12 271–280. 10.1016/j.molcel.2003.08.016 14536067

[B52] Shi Y. Massague J. (2003). Mechanisms of TGF-beta signaling from cell membrane to the nucleus. *Cell* 113 685–700. 10.1016/S0092-8674(03)00432-X 12809600

[B53] Shihab F. S. Bennett W. M. Yi H. Andoh T. F. (2006). Effect of Cyclosporine and Sirolimus on the Expression of Connective Tissue Growth Factor in Rat Experimental Chronic Nephrotoxicity. *Am. J. Nephrol.* 26 400–407. 10.1159/000095300 16926534

[B54] Shihab F. S. Bennett W. M. Yi H. Choi S. O. Andoh T. F. (2004). Sirolimus increases transforming growth factor-beta1 expression and potentiates chronic cyclosporine nephrotoxicity. *Kidney Int.* 65 1262–1271. 10.1111/j.1523-1755.2004.00498.x 15086465

[B55] Simler N. R. Howell D. C. Marshall R. P. Goldsack N. R. Hasleton P. S. Laurent G. J. (2002). The rapamycin analogue SDZ RAD attenuates bleomycin-induced pulmonary fibrosis in rats. *Eur. Respir. J.* 19 1124–1127. 10.1183/09031936.02.00281602 12108867

[B56] Tirnitz-Parker J. E. Olynyk J. K. Ramm G. A. (2014). Role of TWEAK in coregulating liver progenitor cell and fibrogenic responses. *Hepatology* 59 1198–1201. 10.1002/hep.26701 24038142

[B57] Tomei P. Masola V. Granata S. Bellin G. Carratu P. Ficial M. (2016). Everolimus-induced epithelial to mesenchymal transition (EMT) in bronchial/pulmonary cells: when the dosage does matter in transplantation. *J. Nephrol.* 29 881–891. 10.1007/s40620-016-0295-4 27026415

[B58] Tulek B. Kiyan E. Toy H. Kiyici A. Narin C. Suerdem M. (2011). Anti-inflammatory and anti-fibrotic effects of sirolimus on bleomycin-induced pulmonary fibrosis in rats. *Clin. Invest. Med.* 34:E341. 22129924 10.25011/cim.v34i6.15894

[B59] Umemura A. Park E. J. Taniguchi K. Lee J. H. Shalapour S. Valasek M. A. (2014). Liver damage, inflammation, and enhanced tumorigenesis after persistent mTORC1 inhibition. *Cell Metab.* 20 133–144. 10.1016/j.cmet.2014.05.001 24910242 PMC4079758

[B60] van der Poel H. G. (2004). Mammalian target of rapamycin and 3-phosphatidylinositol 3-kinase pathway inhibition enhances growth inhibition of transforming growth factor-beta1 in prostate cancer cells. *J. Urol.* 172 1333–1337. 10.1097/01.ju.0000138829.97838.19 15371835

[B61] Wang B. Ding W. Zhang M. Li H. Gu Y. (2015). Rapamycin attenuates aldosterone-induced tubulointerstitial inflammation and fibrosis. *Cell. Physiol. Biochem.* 35 116–125. 10.1159/000369680 25547416

[B62] Wang P. Liu T. Cong M. Wu X. Bai Y. Yin C. (2009). Expression of extracellular matrix genes in cultured hepatic oval cells: an origin of hepatic stellate cells through transforming growth factor beta? *Liver Int.* 29 575–584. 10.1111/j.1478-3231.2009.01992.x 19323784

[B63] Wang S. Wilkes M. C. Leof E. B. Hirschberg R. (2010). Noncanonical TGF-beta pathways, mTORC1 and Abl, in renal interstitial fibrogenesis. *Am. J. Physiol. Renal Physiol.* 298 F142–F149. 10.1152/ajprenal.00320.2009 19846571 PMC2806113

[B64] Weiskirchen R. (2016). Hepatoprotective and anti-fibrotic agents: it’s time to take the next step. *Front. Pharmacol.* 6:303 10.3389/fphar.2015.00303 PMC4703795 26779021

[B65] Weiskirchen R. Weiskirchen S. Tacke F. (2018). Organ and tissue fibrosis: molecular signals, cellular mechanisms and translational implications. *Mol. Aspects Med.* 10.1016/j.mam.2018.06.003 [Epub ahead of print]. 29958900

[B66] Weng H. Ciuclan L. Liu Y. Hamzavi J. Godoy P. Gaitantzi H. (2007). Profibrogenic transforming growth factor-β/activin receptor-like kinase 5 signaling via connective tissue growth factor expression in hepatocytes. *Hepatology* 46 1257–1270. 10.1002/hep.21806 17657819

[B67] Williams M. J. Clouston A. D. Forbes S. J. (2014). Links between hepatic fibrosis, ductular reaction, and progenitor cell expansion. *Gastroenterology* 146 349–356. 10.1053/j.gastro.2013.11.034 24315991

[B68] Wu Y. Ding Z. Jin G. Xiong Y. Yu B. Sun Y. (2018). Autocrine transforming growth factor-β/activin A-Smad signaling induces hepatic progenitor cells undergoing partial epithelial-mesenchymal transition states. *Biochimie* 148 87–98. 10.1016/j.biochi.2018.03.003 29544731

[B69] Wullschleger S. Loewith R. Hall M. N. (2006). TOR signaling in growth and metabolism. *Cell* 124 471–484. 10.1016/j.cell.2006.01.016 16469695

[B70] Xu X. Dai H. Geng J. Wan X. Huang X. Li F. (2015). Rapamycin increases CCN2 expression of lung fibroblasts via phosphoinositide 3-kinase. *Lab. Invest.* 95 846–859. 10.1038/labinvest.2015.68 26192087

[B71] Xu X. Wan X. Geng J. Li F. Yang T. Dai H. (2013). Rapamycin regulates connective tissue growth factor expression of lung epithelial cells via phosphoinositide 3-kinase. *Exp. Biol. Med.* 238 1082–1094. 10.1177/1535370213498976 23986222

[B72] Yang H. Rudge D. G. Koos J. D. Vaidialingam B. Yang H. J. Pavletich N. P. (2013). mTOR kinase structure, mechanism and regulation. *Nature* 497 217–223. 10.1038/nature12122 23636326 PMC4512754

[B73] Zawel L. Dai J. L. Buckhaults P. Zhou S. Kinzler K. W. Vogelstein B. (1998). Human Smad3 and Smad4 are sequence-specific transcription activators. *Mol. Cell* 1 611–617. 10.1016/S1097-2765(00)80061-1 9660945

[B74] Zhang D. Y. Friedman S. L. (2012). Fibrosis-dependent mechanisms of hepatocarcinogenesis. *Hepatology* 56 769–775. 10.1002/hep.25670 22378017 PMC4087159

[B75] Zhang Y. E. (2009). Non-Smad pathways in TGF-beta signaling. *Cell Res.* 19 128–139. 10.1038/cr.2008.328 19114990 PMC2635127

[B76] Zhu J. Wu J. Frizell E. Liu S. L. Bashey R. Rubin R. (1999). Rapamycin inhibits hepatic stellate cell proliferation in vitro and limits fibrogenesis in an in vivo model of liver fibrosis. *Gastroenterology* 117 1198–1204. 10.1016/S0016-5085(99)70406-3 10535884

