# Supplementary material for: Rapamycin Upregulates Connective Tissue Growth Factor Expression in Hepatic Progenitor Cells Through TGF-β-Smad2 Dependent Signaling
Source: Front Pharmacol. 2018 Aug 8;9:877. doi: 10.3389/fphar.2018.00877 (PMC6092675; doi:10.3389/fphar.2018.00877)

Supplementary Material

Rapamycin Upregulates Connective Tissue Growth Factor Expression in Hepatic Progenitor Cells through TGF-β-Smad2 Dependent Signaling

Yu Wu[1], Wei Wang[1], Xiang-mei Peng[2], Yi He[1], Yi-xiao Xiong[1], Hui-fang Liang[1], Liang Chu[1], Bi-xiang Zhang[1], Ze-yang Ding[1*] and Xiao-ping Chen[1*]

[1]Hepatic Surgery Center, Tongji Hospital, Tongji Medical College, Huazhong University of Science and Technology, Wuhan, Hubei, China

[2]Department of Nephrology, Liyuan Hospital, Tongji Medical College, Huazhong University of Science and Technology, Wuhan, Hubei , China

*** Correspondence:** Ze-yang Ding, E-mail: zyding@tjh.tjmu.edu.cn
Xiao-ping Chen, E-mail: xpchen@tjh.tjmu.edu.cn

1.1 Supplementary Figures

Supplementary Figure 1. Effects of rapamycin on CTGF expression in different cell lines. BRL (A), QSG-7701 (B), HepG2 (C), Hep3B (D), SMMC-7721 (E) and HCCLM3 (F) cells were treated with Rapamycin at indicated concentrations for 6 h. Lysates were subjected to Western blot analysis with antibodies against CTGF. β-actin was used as a loading control.

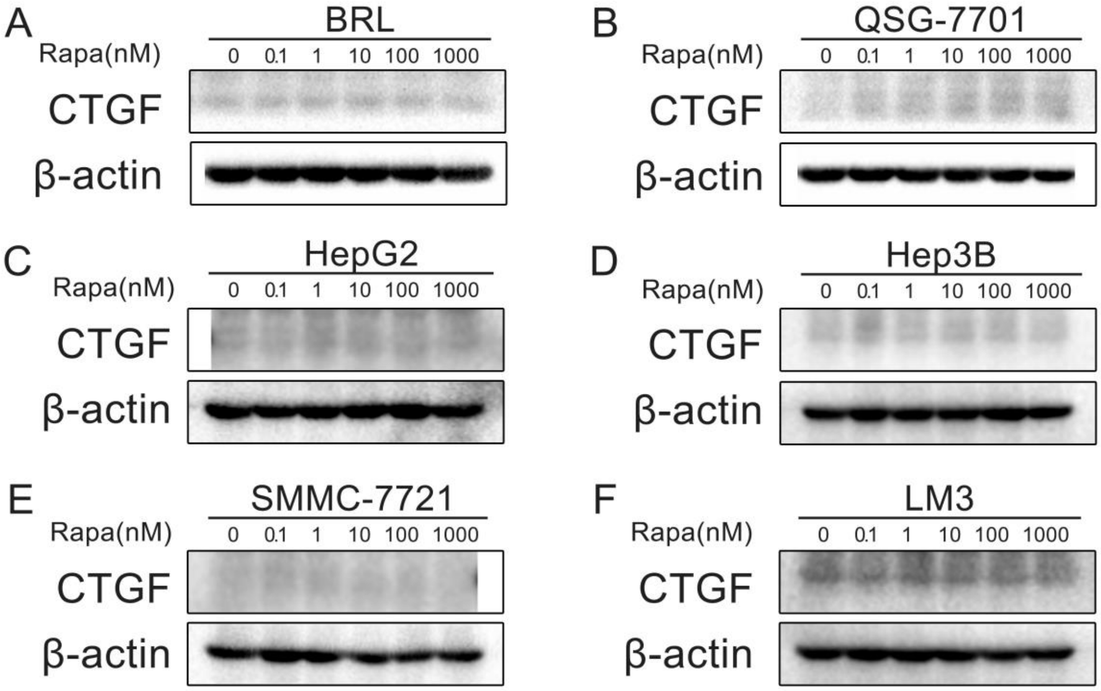

Supplementary Figure 2. Signaling pathway screening after knockdown of mTOR. (A) LE/6shmTOR cell was treated with indicated inhibitors for 6 h. Lysates were subjected to Western blot analysis with antibodies against CTGF. β-actin was used as a loading control.

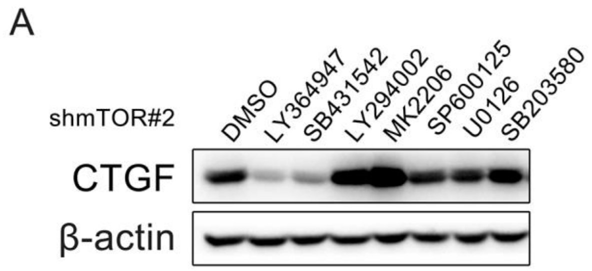

Supplement: Supplementary file 1 [file Presentation_1.PDF]
